# Opportunities and computational challenges in large-scale whole-genome sequencing data analysis

**DOI:** 10.1093/jas/skaf292

**Published:** 2025-08-27

**Authors:** Hafedh Ben Zaabza, Mohammad H Ferdosi, Ismo Strandén, Beatriz C D Cuyabano, Mahesh Neupane, Ignacy Misztal, Daniela Lourenco, Cedric Gondro

**Affiliations:** Department of Animal Science, Michigan State University, East Lansing, MI 48824; Animal Genetics and Breeding Unit, a joint venture between the NSW Department of Primary Industries and Regional Development, University of New England, Armidale, New South Wales 2351, Australia; Natural Resources Institute Finland (Luke), FI-31600 Jokioinen, Finland; INRAE, AgroParisTech, GABI, Université Paris Saclay, 78350 Jouy-en-Josas, France; Animal Genomics and Improvement, Agricultural Research Service, US Department of Agriculture, Beltsville, MD 20705; Department of Animal and Dairy Science, University of Georgia, Athens, GA 30602; Department of Animal and Dairy Science, University of Georgia, Athens, GA 30602; Department of Animal Science, Michigan State University, East Lansing, MI 48824

**Keywords:** computations, genomic prediction, genomic selection, sequence data

## Abstract

Genomic selection has been used in animal breeding for c. 15 yr and continues to be an important tool in predicting genetic merit in livestock populations. The dairy cattle industry was the first to adopt genomic selection, initially based on some 50K single-nucleotide polymorphism (SNP) arrays for thousands of animals. Later advances in genome-scanning technologies have enabled inexpensive genotyping and sequencing, leading to wider adoption, and constantly increasing amounts of genomic data, both as to the number of genotyped animals and variants genotyped per animal. Full sequence data are expected to supersede SNP chips in the coming years. We review the methods and computational approaches used with sequence data and the impact of the methods and model assumptions on genomic prediction accuracy. The modeling, development, and applicability of these methods to sequence data are discussed, as well as the computational resources required. Sequence data should, in principle, provide full information on genetic variability, which should lead to higher prediction accuracy. In practice, there is limited evidence of additional benefit from using sequence data over medium- or high-density SNP panels. This is particularly true for small effective population sizes (*N*_e_) such as cattle populations, where animals within a breed have many common ancestors and thus longer chromosome segments with high linkage disequilibrium accurately trackable with a relatively small number of markers. A population with a small *N* has long haplotype blocks, from 1 to 5 Mb, making it hard to identify causal variants within blocks. However, in major cattle breeds, a medium-density SNP panel is sufficient to tag the blocks themselves, and prediction with large datasets is highly accurate. Clearly, sequence data should not be used directly for genomic prediction, but for identifying putative causal variants to improve the accuracy and stability of subsequent predictions. We show that the best strategy to deal with any large data with high SNP densities is to use only a subset of (important) markers and determine the most appropriate model for exploiting the preselected variants in the genomic evaluation. Novel prediction methods that subset trait-specific informative markers could offer the advantage of using sequence data by potentially linking individuals through underlying functional variants rather than simply through shared haplotype blocks inherited from ancestors. Further research is required to clarify this aspect.

## Introduction

Estimation of breeding values is the cornerstone of animal breeding. These are traditionally estimated from pedigree records and phenotypic data, and, nowadays, genomic information is routinely used as well. The first single-nucleotide polymorphism (SNP) genotype array was developed in 1998 as a joint effort between the Whitehead Institute and Affymetrix, Inc., and contained 1,494 human SNPs (Wang et al., 1998). The first livestock reference genome assembly was for cattle in 2009 (Bovine Genome Sequence and Analysis Consortium et al., 2009), which spearheaded the development of the first bovine SNP array, which became commercially available between 2008 and 2009. This first SNP array contained 54,001 SNPs (Matukumalli et al., 2009) and was developed together with Illumina (San Diego, CA). Although traditional BLUP models were performing well using only pedigree and phenotypic records, after 2009, the SNP information that was becoming available had to somehow be accounted for. Incorporation of this new data source to estimate breeding values required some changes to models and evaluation pipelines—and preferably without completely disrupting the systems already in place in breeding programs. An elegant and seamless adaptation of the traditional BLUP methods was developed by VanRaden (2008), which is now known as genomic BLUP (GBLUP), in which the relationship matrix (**A**) constructed from the pedigree is replaced by a genomic relationship matrix (**G**) that is calculated from the SNP genotypes.

Genotyping costs have decreased significantly from $250 to $25 per sample between 2009 and 2024. Nonetheless, usually only a small percentage of the animals in the pedigree are genotyped (between 5% and 15% in beef cattle). The problem of not having all animals genotyped was solved by a model called single-step GBLUP (ssGBLUP) (Legarra et al., 2009; Aguilar et al., 2010; Christensen and Lund, 2010). The method combines the **G** matrix of genotyped animals with the **A** matrix of ungenotyped animals in a unified relationship matrix **H**. This approach could be more accurate than any other genomic prediction model, as it could make use of the additional available information from ungenotyped individuals (Legarra et al., 2014).

Accuracy gains from genomic models compared to BLUP usually vary between 5% and 40% depending on the trait and population (Lourenco et al., 2015; Tsuruta et al., 2021). Adoption of genomic breeding has greatly increased the rates of genetic gain, and it has been transformational for animal breeding (García-Ruiz et al. 2016); however, the gains reported in real animal populations generally lag those reported in theoretical studies with simulated populations (e.g., Meuwissen et al., 2001). Part of this difference has been attributed to the limited number of SNPs in the current SNP chips, which range from 10K to 800K, mostly within 50K to 100K. Meuwissen et al. (2010) suggested that whole-genome sequence (WGS) data could increase the accuracy of genomic selection as it would mitigate the problem of linkage disequilibrium (LD) decay between SNP markers and causal variants (CVs), and potentially, even the actual CVs could be genotyped. To investigate this, along with other research priorities such as the identification of CVs, the 1000 Bulls Genome Project was established. This consortium aimed to gather WGSs of worldwide cattle (Hayes and Daetwyler, 2019), and while the initial aim was to sequence 1,000 bulls, the latest run (run 9) included 6,191 bulls and cows. In comparison, in the United States, more than 8 million dairy cattle were genotyped with medium-density SNP chips over the last 10 yr (https://uscdcb. com/database-stats/).

With new technological advancements in recent years and plummeting costs, sequencing may soon replace traditional SNP arrays. Technologies like low-pass sequencing (LPS) (short-range and second-generation sequencing) and long-range sequencing (third-generation sequencing) can open new doors to cutting-edge research (Hu et al., 2021). This article aims to discuss the challenges and benefits of whole-genome sequencing data for genomic selection and genome-wide association studies (GWAS).

## Whole-Genome Sequencing Data: From Short-Reads to Long-Reads

Short-read sequencing has a high base-calling accuracy and is a cost-effective platform that is widely used to detect SNPs, single-nucleotide variants, small insertions and deletions (INDELs), and can even be used for de novo genome assemblies (Nguyen et al., 2023). An advantage of short-read sequencing is that the library preparation does not require DNA of very high quality (Nguyen et al., 2023). However, short-reads typically consist of only 100 to 200 bases, which makes them less suitable to detect larger structural variations (SVs), for accurate haplotype phasing and highly accurate genome assemblies, as these benefit from information spanning longer sequence lengths (Van Dijk et al., 2014). Logsdon et al. (2020) pointed out several shortcomings in the use of short-read sequencing data to detect SVs in human genomes: for example, reads of length less than 300 kb, such as those from Illumina’s next-generation sequencing technology, could only detect 30% of the human genome’s SVs, especially the longer ones.

Long-read sequencing is a more recent third-generation sequencing technology that is used by platforms such as Pacific Biosciences High Fidelity (PacBio HiFi) and Oxford Nanopore Technologies (ONT), which enables reads of up to several thousand kb and makes it ideal for performing genome assemblies. Long-read sequencing addresses the limitations of short-reads, albeit at a higher cost and a lower accuracy; although the latter is rapidly approximating the accuracy of short-read sequencing as the technology matures (Logsdon et al. (2020)). The higher cost of long-read sequencing compared to short-read sequencing is still a limiting factor, but sequencing costs are also rapidly coming down. Additionally, the technology allows for highly contiguous genome assemblies, accurate mapping to a reference genome, and longer fully resolved molecular haplotypes.

## Structural Variants

SNPs have long been known to account for a substantial proportion of the phenotypic variation among individuals, either as tag SNPs in LD with the CVs or directly as the CV itself (Stranger et al., 2007). The development of whole-genome scanning technologies together with large-scale community efforts, such as the 1000 Bull Project, has raised a growing interest in understanding the role that structural variants play as a source of genetic and phenotypic variation. An interesting and valid point by Alkan et al. (2011) is that SVs cause larger differences in genomes than differences between single-base pairs.

An SV is conventionally defined as a DNA sequence ranging from 50 base pairs to several mega-bases (Weischenfeldt et al., 2013; Collins et al., 2020; Nguyen et al., 2023), which exhibits a change in copy number variation (CNV) (deletions, insertions, and duplications), orientation (inversions), or chromosomal location (translocations) between individuals (Alkan et al. (2011); Escaramís et al., 2015). SVs can be grouped into various mutational categories, including balanced rearrangements occurring without the corresponding dosage alterations (inversions and intrachromosomal trans-locations), and unbalanced rearrangements involving gains or losses of DNA (CNVs, comprising deletions, insertions, and duplications) (Collins et al., 2020). Because of their size and abundance in the genome, SVs possess relevant mutational power that influences genomic evolution, gene functions, and numerous common and rare human diseases (Collins et al., 2020; Weischenfeldt et al., 2013).

In humans, SVs are associated with various diseases, including cancer, Alzheimer’s disease (Macintyre et al., 2016), cystic fibrosis (Hayden et al., 2008), Charcot-Marie-Tooth disorder (Pyromali et al., 2021), autism (Hedges et al., 2012), and Williams–Beuren syndrome (Gheldof et al., 2013). SVs are also known to influence gene expression (Gheldof et al., 2013), gene regulation (Spielmann and Mundlos, 2013), and the 3D structure of DNA (Spielmann et al., 2018), which affects phenotypes. Partially overlapping deletions in the 22q11 gene have been shown to be associated with the DiGeorge syndrome (DGS) and the velo-cardio-facial syndrome (Maynard et al., 2002). Laugsch et al. (2018) demonstrated that the branchio-oculo-facial syndrome is caused by an inversion, which disconnects TFAP2A from its enhancers. The impact of genomic SV on phenotypes, including human diseases, is well discussed in Weischenfeldt et al. (2013).

In animals, SVs are found to impact growth traits in yaks (Wang et al., 2023), olfaction, coat colors, carcass, and skeletal traits, as well as meat quality in pigs (Giuffra et al., 2002; Nguyen et al., 2012; Zong et al., 2023; Kwon et al., 2024), the muffs, beard and peacomb phenotype in chicken (Wright et al., 2009; Guo et al., 2016), the polled genetics in beef cattle (Mueller et al., 2021), polycerate, supernumerary nipples, ear size, litter size, coat colors, and tail traits in sheep (Norris and Whan, 2008; Cumer et al., 2021; Salehian-Dehkordi et al., 2021), and could affect testis tissue, immune response, olfactory functions, cell proliferation, epidermal differentiation, skin barrier function, and resistance to bovine tuberculosis and glucose metabolism in beef muscles (Zhou et al., 2022; Bhati et al., 2023). To enhance the understanding of SVs in cattle genomes, the Bovine Long Read Consortium (BovineLRC), an international collaboration with a focus on leveraging long-read sequencing technologies, was established. Along with the improved SV detection in cattle, long-reads also help in better resolution of repetitive regions, creation of high-quality reference genome, haplotype phasing, enhanced isoform resolution in transcriptomics, and better detection of epigenetic modifications (Nguyen et al., 2023). It is worth noting that among the most famous SVs in bovines associated with the polled phenotype are the Celtic and Friesian polled SVs. Both variants result in the same (polled) phenotype, but they differ in the exact sequences and altered regulatory regions. The Friesian polled allele is associated with an 80-kb DNA duplication on BTA1 (Rothammer et al., 2014), whereas the Celtic polled mutation is a 202 base pair (bp) insertion–deletion event on BTA1 and involves the regulation of a long inter-genic noncoding RNA (Wiedemar et al., 2014). Although these mutations are different, both include the RXFP2 gene, which is crucial for horn development (Wiedemar et al., 2014).

CNVs are among the largest genetic variants and have unique functional consequences. Several studies have reported that CNVs imply more genomic sequence alterations than SNPs and can have stronger effects on gene expression and gene function, particularly gene dosage, disruption of coding sequences, or alteration of gene regulation (Zhang et al., 2009; Xu et al., 2014; Liu et al., 2010). While CNVs can be in LD with neighboring SNPs (Conrad et al., 2006; Hinds et al., 2006), a significant fraction of CNVs is not easily tagged by SNPs (Liu et al., 2010). Those CNVs remain elusive and frequently fall in genomic regions sporadically covered by SNP arrays and are thus not genotyped (Liu et al., 2010). Studies have suggested that investigating the genome for both CNVs and SNPs could be an effective way to obtain a more mechanistic insight into the origins of phenotypes, including human diseases (Liu et al., 2010).

In livestock, the genotyping array (e.g., VanTassell et al., 2008; Matukumalli et al., 2009) heralded in the genomic selection era and became the de facto standard for genomic prediction; however, the use of SV markers in livestock breeding is still incipient (Nguyen et al., 2023).

With their accompanying pros and cons, both short- and long-read sequencing platforms are used for the identification of SVs. Tools such as NGMLR and Sniffles have been developed to detect SVs (Sedlazeck et al., 2018), but identification remains challenging. Another challenge that arises in analyzing and identifying SVs in livestock genomes has to do with the reference genome that is usually based on a single breed, for example, the Bos taurus genome derived from a single Hereford cow (Bovine Genome Sequence and Analysis Consortium et al., 2009), which differs significantly from other breeds, such as Brahman (Leonard et al., 2022). To address this problem, breed-specific reference genomes have been used, but these are expected to be soon superseded by the various livestock species-specific pan-genomes that are currently being developed.

In summary, SVs are known to play a crucial role in influencing phenotypes in both humans and animals. The advent of long-read sequencing technologies like ONT and PacBio has increased the potential for improved reliability and accuracy of SV detection, which should allow their adoption in genomic selection programs. This holds promise to increase the accuracy of genomic prediction in the livestock industry, although the development of new methodologies and software is still necessary to utilize this information effectively.

## Low-Pass Sequencing

Low-pass genome sequencing is characterized by a low- coverage approach with a depth of less than 1× in products like SkimSEEK (Corporation, 2021), and due to its low coverage, it results in the identification of one haplotype with random parental origin. This may pose challenges for deterministic analysis, particularly in parentage or duplicate geno-type determination based on opposing homozygotes (Ferdosi and Boerner, 2014). In fact, LPS can be viewed as single- allele genotyping, which leads to ambiguity in assessing heterozygosity or homozygosity of the allele. This issue can be addressed using low-pass and high-depth sequencing methods, such as InfiniSEEK (Neogen, 2023).

According to Li et al. (2021), LPS offers several distinct advantages in comparison to SNP arrays: (1) LPS eliminates ascertainment bias regarding the variants or sites on the genome under investigation; (2) it facilitates the discovery of novel variations at the sample or population level, evidenced by studies such as Tran et al. (2020) and Liu et al. (2010); (3) it allows for cost-effective sequencing by enabling the multiplexing of a large number of samples in massively parallel LPS efforts; (4) it provides the ability to fine-tune the accuracy of a sample’s imputed genotypes by adjusting the target coverage, thus affording greater flexibility in designing experiments within practical logistical or financial constraints; (5) when employed at approximately 0.5× coverage with subsequent imputation, LPS can enhance the effectiveness of GWAS compared to using platforms like the Illumina Global Screening Array. This approach has been shown to improve the accuracy of polygenic risk scores, that is, the additive effect of genes on the phenotype, as demonstrated by Li et al. (2024); (6) at 1× coverage it can be cheaper than SNP arrays (Rubinacci et al., 2021); and, finally, (7) it can improve the imputation accuracy by selective coverage of the genome.

## Low-Pass Sequence Imputation

Imputation of missing genotypes is an initial step in genomic analysis in most studies. This is motivated by two key factors. First, many algorithms assume complete genotype availability and omit individuals with any missing genotypes during implementation for simplicity. Second, because imputation accuracy tends to be high in typical scenarios, using imputed genotypes may improve the accuracy of subsequent analyses.

Dosage data provide a probabilistic measure of genotypes, reflecting the probability-weighted sum of alleles. This is more informative than “hard” calls, which assign a definite geno-type without capturing the underlying uncertainty (Browning and Browning, 2009; Marchini and Howie, 2010). The use of dosage data in association studies has shown to increase their statistical power, enabling the detection of associations that might be missed when using hard calls (Marchini and Howie, 2010; Zhou and Stephens, 2014). Particularly for low- frequency variants, the use of dosage accounts for the confidence level of genotype calls and reduces the potential bias from the misclassification seen in hard calls (Browning and Browning, 2009; Hayes et al., 2009). However, the continuous nature of dosage data requires more computational power for storage and analysis as compared to hard calls (Das et al., 2016). Handling of larger datasets with dosage values also demands more sophisticated algorithms and computational infrastructure (van Binsbergen et al., 2014). Dosage data can be less intuitive due to their probabilistic nature, which may complicate interpretation and application (Hayes et al., 2009). Some of the existing analysis software does not support the use of dosage data as readily as hard calls, thus limiting their utility (Marchini and Howie, 2010). The effectiveness of using dosage data depends heavily on the accuracy of the imputation process and can compromise the reliability of genomic analyses when imputation accuracy is low (van Binsbergen et al., 2014; Das et al., 2016).

GLIMPSE 2 is a novel high-accuracy imputation algorithm based on the GLIMPSE model for low-coverage whole- genome sequencing (Rubinacci et al., 2021). It is a program of choice and a highly efficient tool specifically designed for the imputation and phasing of LPS data when large reference panels are available. Its role in imputation is to predict the most likely genotype for a given individual based on the genotypes of other individuals and the patterns observed in the reference panel. Several studies have shown moderate to high imputation accuracy in LPS compared to SNP genotype arrays (Snelling et al., 2020; Buckley et al., 2022).

Calling the variants is also an important factor for the imputation of LPS. DeepVariant is a highly accurate variant calling tool based on deep learning (convolutional neural networks). It outperforms traditional tools such as the Genome Analysis Toolkit (GATK) (O’Connor and van der Auwera, 2020) in terms of accuracy, especially in regions with complex variants (Poplin et al., 2018). However, the overall computational cost of DeepVariant is higher, and it is slower on general-purpose CPUs but benefits greatly from the use of Graphics Processing Units (GPUs). It is worth noting that DeepVariant does not require reprocessing the entire dataset when adding new individuals, unlike tools such as GATK, which rely on joint- genotyping strategies for cohort-level variant calling.

The DeepVariant (Poplin et al., 2018) algorithm is reported to be a better variant caller than GATK (O’Connor and van der Auwera, 2020) and leads to higher imputation accuracy using the GLIMPSE model. High imputation accuracy was obtained with the breed of the haplotype included in the reference haplotype library, whereas without the haplotype of a specific breed in the reference population, imputation accuracy for LPS was low (O’Connor and van der Auwera, 2020). Indeed, accurate imputation of data in a common cattle breed can be achieved by using medium-sized, breed-specific haplo-type reference panels, as well as large, multibreed haplotype reference panels (Lloret-Villas et al., 2023).

Human genome studies show that LPS is a good option for statistical and population genetics applications. If more accurate information is needed, the UK Biobank sequence data can be used to improve LPS imputation accuracy. It is anticipated that the distinction between low-coverage and high-coverage WGS will diminish over time due to the ongoing expansion of comprehensive reference panels gathering a greater range of human haplotype diversity (Rubinacci et al., 2023). Using LPS at 0.4× provided higher imputation accuracy and performance than a genotyping array; however, this was not true for some specific genome regions. This issue can be solved by combining LPS and high-depth sequencing (Wasik et al., 2021). In a study of the canine genome, it was shown that although LPS can accurately impute missing genotypes, it may not be suitable for haplotype analysis because it may affect the accuracy of phasing. For high phasing accuracy, the minimum depth should exceed 20× (Wragg et al., 2024). However, it should be noted that the sample size in this study was small.

## Experiences With Sequence Data in Livestock

It is well known that the accuracy of predicting breeding values based on low- and medium-density SNP array-based is partially due to the close additive genetic relationships between animals in the training and validation populations. SNPs capture not only effects due to LD between SNP markers and quantitative trait loci (QTL), but also the additive genetic relationships, and thus the accuracy is not zero even for weak LD because the accuracy is gained by better relationships (or indirectly by estimating chromosome segments that segregate from related animals), and not only by linkage to QTL. As a result, the genomic prediction accuracy does not persist across generations but decreases markedly over time as large chromosome segments break up because of recombination.

Based on the idea that the accuracy of genomic prediction depends on two factors: (1) the proportion of genetic variance explained by SNP markers, and (2) the precision with which the SNP solutions are computed, Goddard (2017) mathematically showed two pathways for achieving a genomic selection accuracy of 100% in Holstein dairy cattle. By hypothesizing that CVs have properties similar to the SNP markers, the amount of LD between CVs and SNPs would then be equal to that between SNPs themselves. Thus, the variance explained by SNPs can be written as $\frac{m}{m+Me}$, where *m* is the number of SNPs and *M_e_* is the number of independent chromosome segments in the genome. Goddard (2017) showed that, in the scenario of 3,000 *M_e_* and 50,000 SNPs, the variance explained by SNPs is equal to 94%. Nevertheless, lower LD between CVs and SNPs than between SNPs themselves might still be possible. For example, CVs often have a lower minor allele frequency (MAF) than SNPs. As a result, the variance explained by SNPs is smaller than in the above scenario (94%).

Goddard (2017) also demonstrated that the accuracy with which SNP marker effects are estimated depends on the parameter θ =* T × h*^2^/*M_e_*, where *h*^2^ is the heritability of the trait and *T* is the number of individuals in the training population. If *h*^2^ = 0.3, *M_e_* = 3,000, and *T* = 100,000, genomic prediction accuracy would be 95%, assuming that SNPs explain 100% of the genetic variance. Increasing *T* to 1,000,000 would lead to genomic prediction accuracy of 100%. In practice, this is different. According to Gianola (2017), predictions will never be 100% accurate because of wrong model assumptions, even with large data. Cesarani et al. (2022) computed ssGBLUP genomic predictions with around 3.9M genotyped animals, and the maximum accuracy was 0.88.

Theoretically, using WGS data instead of dense SNPs would be expected to substantially improve genomic prediction accuracy because the CVs would actually be in the data being analyzed (Goddard, 2017). Conceptually, using WGS data would have at least three advantages compared to SNP chip genotypes: (1) the impact of the recombination mentioned above could be eliminated because predictions would be based on causal mutations instead of SNPs (Macleod et al., 2014), potentially improving genomic prediction accuracy over generations; (2) because the SNPs selected for the 50K SNP array have a relatively high MAF, they are less likely to be in high LD with CVs which are more likely to have a low MAF, especially in the case of QTL that have been under negative selection, for example, for disease and fertility traits (Hayes et al., 2014; Macleod et al., 2014); (3) genomic prediction accuracy for less represented breeds, that is, breeds with a smaller reference population size, can be improved by combining data from multiple breeds.

Hayes et al. (2014) pointed out that using sequence data for genomic prediction is, in fact, a QTL mapping issue. A significant benefit of the multibreed reference population is that causative mutations can be mapped more accurately because LD across breeds is lower than within breeds (Hayes et al., 2014). Extensive studies have focused on using sequence data for genomic predictions in livestock. Their results have generally shown either no gain or only limited gain in genomic prediction accuracy (Hayes and Daetwyler, 2019; Misztal et al., 2020).

Several researchers turned to simulated sequence data to explore the accuracy of genetic predictions (Meuwissen and Goddard, 2010; Clark et al., 2011; Druet et al., 2014; Macleod et al., 2014) because WGS data for large numbers of animals were not available in the previous decade. The developed genomic prediction models using WGS data can be grouped into two main classes: (1) Bayesian nonlinear models based on the a priori assumption that only a few SNPs are causative, that is, that a large proportion of loci have no effect (Meuwissen et al., 2001; Meuwissen and Goddard, 2010), and (2) BLUP models where all SNPs are assumed to have an effect drawn from the same normal distribution (VanRaden, 2008).

In the following, we review the results of using WGS data, whether in simulated or real populations. Meuwissen and Goddard (2010) simulated WGS data on one chromosome of 1 Morgan and found a substantial increase in genomic prediction accuracy compared to a standard SNP chip. The authors tested different scenarios—3QTL vs. 30QTL per Morgan, and causal SNPs included vs. causal SNPs excluded, and reported very high genomic prediction accuracy, approaching 1, even when training and test data were 10 generations apart. Druet et al. (2014) used sequence data simulated based on a real Belgian Blue cattle pedigree and reported that genomic prediction accuracy relative to using the SNP panel was strongly dependent on the allele frequency distribution of causative mutations affecting the trait. When causative mutations had a low MAF (<1%), an increase of up to 30% was observed, but when QTL allele frequencies followed the same distributions as the other variants, sequence data only slightly improved genomic prediction accuracy (<2%) over SNP arrays. Brøndum et al. (2015) proposed an approach that used the 15 most significant QTL identified from GWAS-imputed sequence data for Nordic Holstein, Nordic Red dairy cattle, and Danish Jersey. For each breed, these QTL were incorporated with 3 to 5 markers to tag each QTL to a regular SNP chip. Including the selected QTL in the SNP panel yielded a gain in genomic prediction accuracy of ~3% for production traits in Nordic Red dairy cattle, ~4% in Nordic Holsteins, and ~5% in French Holsteins.

VanRaden et al. (2017), in an approach similar to Brøndum et al. (2015), combined sequence variants for almost 444 Holstein animals and high-density genotypes for 26,970 progeny- tested Holstein bulls imputed using findhap software. The authors reported that using multiple regression to select 16,648 sequence variants (candidate SNPs) with the largest estimated effects from the WGS and adding them to 60,671 SNPs increased genomic prediction accuracy by about 3%. Macleod et al. (2014) investigated the benefits of sequence data for genomic prediction by examining two scenarios: (1) a population with ancestrally large effective population size (*N*_e_) that was reduced to a small *N*_e_, and (2) a population with a large *N*_e_ and low LD, mimicking human or outbred plant populations. The authors showed that using sequence data under scenario 1 permitted a 22% gain in genomic prediction accuracy compared to high-density SNP arrays ~600K. They further demonstrated that populations with high *N*_e_ and low LD could benefit considerably from the use of sequence data, and that the benefits could be even greater for traits under long-term negative selection. They also emphasized that the benefits of sequence data highly depended on the statistical method of analysis. Later, we will explore the main statistical methods applied in genomic prediction with sequence data.

## Use of Whole-Genome Sequence Data in GWAS

The use of WGS data in GWAS for livestock can increase resolution and power of genetic analyses. Incorporating WGS data facilitates the identification of CVs with greater accuracy compared to array-based genotyping, which is limited to preselected markers. Also, the use of WGS in GWAS enables the detection of rare and low-frequency variants that contribute to phenotypic diversity but are hard to detect with traditional genotyping methods (Meuwissen et al., 2001). The success of WGS trait analysis depends on various factors such as effect size, sample size, confounders, LD, and statistical tools Korte and Farlow (2013).

This discovery of new genetic variants from WGS GWAS helps in a better understanding of the genetic basis of economically important traits in cattle, which can enhance breeding strategies by incorporating those newly found markers into genomic selection programs. A study conducted by Daetwyler et al. (2014) utilized WGS data from 234 bulls to identify genetic variants associated with milk production and quality traits. It reconfirmed the association of the *DGAT1* gene on chromosome 14 with milk fat content in dairy cattle. Additionally, this study found new low-frequency variants on chromosome 20 associated with protein yield and other production traits. These variants were not detected in prior studies using medium- or high-density SNP arrays, demonstrating the ability of whole-genome sequencing to reveal rare genetic variations that contribute significantly to complex traits.

While WGS can enhance the resolution of GWAS, there are many instances where it has not yielded additional significant insights beyond those previously found with traditional geno-typing arrays. For example, certain cattle fertility studies using WGS data for GWAS did not yield additional significant variants beyond those already identified using high-density SNP arrays. This can be attributed to the highly polygenic nature of these traits, where no single or small set of variants has a large enough effect to be discerned even with increased data resolution (Forutan et al., 2024). Some GWAS using WGS data in dairy cattle to find associations with milk yield and composition traits have not consistently identified novel major effect loci compared to prior studies using high-density SNP arrays. The marginal improvement in the identification of CVs has often been limited by the LD in cattle populations, which makes fine mapping challenging. This redundancy means that for certain well-studied traits, WGS did not necessarily offer significant new insights over traditional genotyping methods (Hayes and Daetwyler, 2019; MacLeod et al., 2016). Traits heavily influenced by environmental interactions, such as stress resilience in livestock, have shown limited additional findings through WGS-based GWAS. The complexity of the genetic architecture of these traits, along with significant nongenetic variance, can overshadow the potential benefits of WGS in revealing new loci (Forutan et al., 2024). In livestock, most of the current WGS GWAS studies are based on imputation of medium- or high-density genotypes to WGS, so accuracy of imputation and reference population is crucial (Hayes and Daetwyler, 2019). Although WGS has expanded the potential for GWAS in many areas, its added value is sometimes constrained by factors such as the genetic architecture of traits, environmental influences, imputation accuracy, and the limitations imposed by population LD structure.

Integrating WGS data in GWAS facilitates a more thorough understanding of the genetic architecture underlying complex traits. It supports the exploration of pleiotropy and the interactions between multiple loci (epistasis), providing deeper insights into how genetic networks influence phenotypes in livestock. For example, Daetwyler et al. (2014) demonstrated that WGS data can identify novel SNPs, and SVs linked to traits that impact productivity and adaptability. WGS-based GWAS aids in more effective marker-assisted selection and genomic selection strategies and enhances our understanding of the functional implications of genetic variation, helping with more targeted breeding approaches that align with different livestock management goals.

## Using GBLUP Methods With Sequence Data

VanRaden (2008) introduced statistical methods for genomic predictions using both linear and nonlinear genomic models. For linear model predictions, **A** is replaced by **G**, which assumes an equal and independent distribution of SNP markers, that is, all SNPs have non-zero but small effects that are normally distributed. The elements of **G** are the fraction of DNA shared between individuals, whereas **A** is the expectation of these fractions (Goddard et al., 2011). Because the genomic relationship matrix is commonly referred to as **G**, the model is called GBLUP. The equivalence between GBLUP and SNP-BLUP models has been demonstrated with GBLUP and SNP-BLUP models (Habier et al., 2007; Strandén and Garrick, 2009; VanRaden et al., 2009), even when a residual polygenic effect (RPG) is included (Liu et al., 2016; Ben Zaabza et al., 2020).

Several studies have shown that the accuracy of GBLUP predictions is not sensitive to the number of QTL (Daetwyler et al., 2010; Goddard et al., 2011). Goddard et al. (2011) investigated the accuracy of GBLUP for fat percentage, which is known to be controlled by a gene of major effect, namely *DGAT1*. The authors reported that despite the departure from the normal distribution assumption, GBLUP performed reasonably well. They also showed mathematically the importance of variation in **G** in determining the genomic prediction accuracy. Furthermore, Goddard et al. (2011) noted that the relationship between two individuals is, in fact, an average over many independent chromosome segments *M* as follows: *M_e_* = 2*N_e_Lk*/log(*N_e_L*), where size, *N_e_* is the effective population *L* is the average length of a chromosome in Morgans, and *k* is the number of chromosomes. Based on the above formula for *M_e_*, if *N_e_* is large, common ancestors are likely to be in the distant past, and so recombination will have broken the chromosomes up into many small fragments, which will join together independently. Thus, provided that the relationship is inversely proportional to *M_e_* segments, the larger the *N_e_*, the smaller the variation in the relationship.

A large number of SNP markers to achieve high genomic prediction accuracy is needed in populations with large *N_e_*, such as humans or outbred plants. This can be demonstrated with two formulas: (1) the amount of total variance explained by SNP markers = *m*/(*m* + *M_e_*), where m is the number of SNP markers, and (2) *r*2 = 1/(2 + 4*N_e_c*), where *r*2 is the LD between SNP markers and *c* is the genetic distance between two SNPs in Morgans. It can be seen that as *N_e_* increases, the LD between SNPs and QTLs decreases, indicating the need for a large number of SNPs, where the use of sequence data would be beneficial. On the other hand, for populations with small *N_e_* (~100), such as the Holstein breed, the number of independent chromosome segments *M_e_* is small, and, thus, the variation in the relationships between individuals is high, so that the LD between SNP markers and QTLs will be high. Therefore, only a few SNP markers are required to explain a large proportion of the total variance.

**Figure 1.**
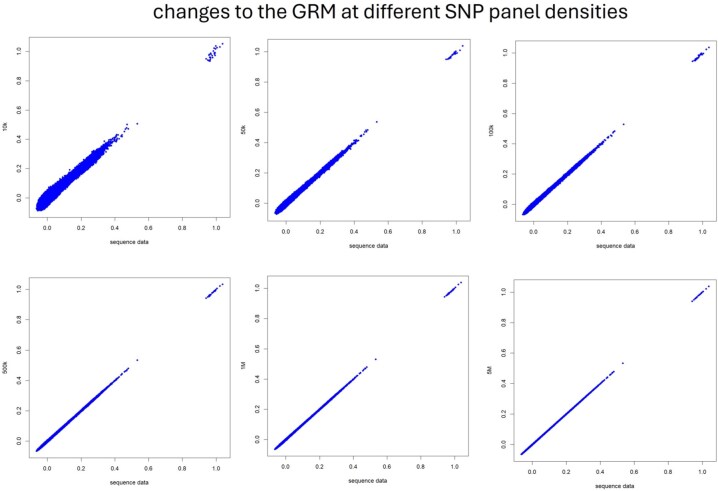
Changes to the genomic relationship matrix **G** at different SNP panel densities. Increasing the number of SNPs from 10,000 to 50,000 slightly improved the agreement between real sequence and the SNP chip. However, raising the number of SNPs beyond 50,000 did not increase the agreement between **G** elements from real sequence data and SNP panel densities larger than 50K.

Figure 1 shows a scatterplot between the elements of G using real sequence data vs. different SNP panel densities. Increasing the number of SNPs from 10,000 to 50,000 slightly improved the agreement between real sequence and the SNP chip. However, raising the number of SNPs beyond 50,000 did not increase the agreement between G elements from real sequence data and SNP panel densities larger than 50K. Similar results have been reported by Goddard et al. (2011). The authors pointed out that the widespread use of artificial insemination in some cattle breeds has caused a dramatic reduction in Ne because one bull can sire many daughters. As a result, genomic prediction accuracy using GBLUP models for cattle data reached an asymptote with only 10,000 SNPs, whereas for human populations, 300,000 SNPs are needed.

It is worth noting that achieving high genomic prediction accuracy for multiple breeds requires a larger number of markers to cover all the variation. In fact, the variation in relationships between breeds is small, and the LD between breeds is limited. In a study of genomic prediction in multiple breeds, Brøndum et al. (2015) found significant improvement from the use of sequence data compared to low- and high-density chips in a multibreed dairy population. Generally, the GBLUP infinitesimal model performs well due to its capability to track the polygenic nature of complex traits, that is, traits controlled by many QTLs, each with small to moderate effects. In this scenario, models like GBLUP, which assume a normal distribution of SNPs, can perform as well as or even better than nonlinear models such as Bayesian models, particularly for populations with a small *N_e_* and high LD, common to many livestock species such as cattle.

The most attractive approach in the analysis of sequence data consists of identifying the CVs and discarding the remaining non-CVs from the prediction model equation. However, GBLUP assumes that all variants have an effect and are equally important. Thus, it is not surprising that GBLUP showed only a small or no gain from using sequence data compared to medium- and high-density chips for domesticated livestock. However, the decay of genomic prediction accuracy over generations, due to the decrease in LD, could be overcome by retraining the prediction model and repeated phenotyping. Misztal et al. (2020) noted that an increase in the size of the genotyped reference population has a greater impact on prediction accuracy than an increase in the number of SNP markers.

## Bayesian Methods for Sequence Data Analyses

In the Bayesian approach, prior information about the distribution of the SNP marker effects can be incorporated into the model. According to Meuwissen et al. (2001), the assumption in models like GBLUP that all SNPs have the same variance seems unrealistic, whereas the assumption that variance can differ between loci seems more realistic. Different Bayesian methods used in association analysis differ only in the prior density specified for the SNP effects. Gianola et al. (2009) named/termed the different Bayesian methods by “Bayesian alphabet” to indicate the expanding letters of the alphabet of the different Bayesian linear regressions currently available.

Gianola and Fernando (1986) stated that Bayesian methods naturally take into account for uncertainty all unknown parameters in a model and all known information in a model. The power and flexibility of Markov chain Monte Carlo allow almost any parametric model to be used in Bayesian inference. Bayesian methods used in association analyses differ only in the specified prior density for the SNP effects while sharing the same sampling model (Gianola, 2013). Gianola et al. (2009) and Gianola (2013) raised questions and provided a critical view regarding the influence of prior assumptions, mainly those developed in Meuwissen et al. (2001), on estimates of marker effects and, particularly, on genomic prediction outcomes. The most common prior distributions in SNP marker models were classified by de los Campos et al. (2013) into four main classes as follows: (1) Gaussian priors: where all SNPs are included in the association model and all SNPs have equal variance; this model is known as Bayesian ridge regression; (2) thick-tailed priors; (3) spike-slab priors; and (4) BayesR, which uses a mixture of priors. The main challenge when dealing with sequence data for genomic predictions is to choose a proper prior density.

Bayesian methods are reported to have better predictive ability than GBLUP methods when using sequence data (Meuwissen et al., 2001; Meuwissen and Goddard, 2010; Ober et al., 2012; Wimmer et al., 2012). Meuwissen and Goddard (2010) compared the BayesB and the standard GBLUP methods under two scenarios: with 3-QTL and 30-QTL data. BayesB delivered better prediction accuracy than GBLUP in both scenarios: 0.826 vs. 0.491 for 30-QTL data, and 0.973 vs. 0.503 for 3-QTL data. Clark et al. (2011) also compared genomic prediction accuracy using sequence data with Bayesian and GBLUP methods in two populations. Both methods were tested under four scenarios: 100, 1,000, 10,000, or >10,000 QTL. The obtained prediction accuracies using BayesB (GBLUP) were 0.87 (0.58), 0.67 (0.60), 0.58 (0.58), and 0.54 (0.55) for the four scenarios, respectively.

Bayesian methods have been unable to show superiority over GBLUP when used with real data, unlike in the simulated data (Clark et al. (2011); Ober et al., 2012). In fact, simulation studies may favor Bayesian methods by having very few QTL with large effects. Indeed, real data are believed to contain more QTL than simulated data, so the assumption that all SNPs have effects fits well. This was clearly demonstrated by Clark et al., 2011, as shown above: the GBLUP method is independent of the number of QTL and distribution effects, that is, GBLUP weights all effective segments equally. In contrast, BayesB attempts to find chromosome segments with large effects. It is important to note that a small *M_e_* is not necessarily an advantage for Bayesian models unless *M_e_* is much larger than the number of QTLs. Daetwyler et al. (2010) reported that in such a scenario, BayesB is relatively more accurate than GBLUP.

Overall, Bayesian approaches have higher predictive ability than GBLUP only when the number of QTL is lower than *M_e_*, and the difference in predictive ability between the two approaches decreases as the number of QTL increases. However, it is worthwhile noting that differences between methods become small if and only if perfect prediction accuracy is achieved, which is not relevant in practical situations where perfect accuracy is far from being reached.

In livestock breeds such as Holstein cattle, *M_e_* is around 15,000 (Pocrnic et al., 2016a), and the number of QTL is less likely to be low. Thus, simulating data with a small number of QTL seems unrealistic. This leads to the following question: Will the availability of more sequence data increase genomic prediction accuracy?

## Using Large Sequence Data for Genomic Predictions

Jang et al. (2023a, 2023b) used preselected variants from sequence data for ssGBLUP genomic predictions in single-and multibreed pig populations. The number of animals with sequence information ranged from 29K to 104K in the single line and was equal to 206K in the multiline. Accuracy increases were minimal, mostly around 0.01. Using the same data, RosFreixedes et al. (2022) showed that accuracies increased from 0.55 to 0.59 when moving from a chip with 40K SNP to 40K selected sequence variants with BayesR (Moser et al., 2015). With ssGBLUP, accuracies increased from 0.59 to 0.60 in the same scenario (Jang et al., 2023b). Although there was a considerable increase with BayesR, the baseline accuracy was greater with ssGBLUP. Using ssGBLUP (Fragomeni et al., 2019) and BayesA (VanRaden et al., 2017) with sequence- selected SNPs for almost 27K Holstein bulls, no increase in accuracy was observed in the former, but a small increase was observed in the latter. Because ssGBLUP includes all available information for genotyped and non-genotyped animals, unlike Bayesian methods or pure GBLUP (i.e., only genotyped animals with phenotypes), any prior assumptions about SNP effects are overwhelmed by the data (Fragomeni et al., 2019; Jang et al., 2023a), which explains the lack of major gains in ssGBLUP.

Although the assumption of GBLUP and ssGBLUP is that SNPs explain the same proportion of variance, it is possible to weight SNPs differently in these methods (Wang et al., 2012). Lourenco et al. (2017) showed that, for less polygenic traits, adding weights for SNPs has a greater impact in small genotyped populations. Recently, Chegini et al. (2025) demonstrated that the use of trait-specific marker weights improved prediction reliability in the analysis of udder health traits of Nordic Red and Jersey cattle populations. As the number of genotyped animals increases, weighting SNPs becomes unimportant. Using Bayesian methods or weighting SNP in GBLUP-based methods has a similar effect. Karaman et al. (2016) showed that the difference in accuracy among BayesB, BayesC, and unweighted GBLUP was large when the number of genotyped individuals was small but approached zero when the size of the population increased.

Several factors can affect our ability to use sequence data for genomic predictions. One is the computational resources, which will be discussed later. As sequence data is highly redundant, selecting variants based on GWAS is a logical approach. However, identifying these variants is challenging; the number of significant SNPs is often low (Ros-Freixedes et al., 2022). Jang et al. (2023c) investigated the relationship between sample size in GWAS, the amount of information for each sample, and *M_e_* (i.e., independent chromosome segments). According to Pocrnic et al. (2016b), *M_e_* can be approximated by the number of largest eigenvalues that explain 98% of the variance in **G** (EIGEN98), which gives an idea of the dimensionality of genomic information. However, the number of eigenvalues—and consequently, *M_e_*—would vary depending on the threshold used for the variance explained. Jang et al. (2023c) found very few causative variants in populations with large *N_e_* (200) when the sample size was equal to EIGEN98 and each sample had one phenotype. When each sample had many progeny records, mimicking genomic EBV (GEBV) reliabilities of 0.99, many of the 2,000 simulated causative variants were significant. Very few causative variants were identified for a population with *N_e_* equal to 20, even though the genotyped animals had near-perfect accuracy. With such a small *N_e_*, many more samples with a lot of information are needed.

In their recent study, Pocrnic et al. (2024) examined the distribution pattern of estimated SNP effects proximal to causative variants and their detectability depending on *N_e_* in simulated populations. This pattern was named the QTL profile. Using single-step GWAS on simulated datasets under various scenarios of *N_e_* sizes and genotyped animals with phenotypes, the authors showed that the Manhattan plots are composed of four components, namely QTL, QTL profile, relationships between individuals, and noise due to the estimation error. The QTL profile was found to be similar to the curve of expected pairwise linkage disequilibrium. The authors observed that the QTL profile is a function of *N_e_*. Indeed, for populations with small *N_e_*, QTL profiles are wide and relationships are strong. In contrast, for populations with large *N_e_*, the width of QTL profiles is narrow and relationships between individuals are weak. Pocrnic et al. (2024) further demonstrated that there is some confounding between QTL profiles and signals due to relationships, which causes limitations of resolutions of GWAS and poor discovery rates compared to populations with large *N_e_*. The authors argued that for populations with small *N_e_*, a high prediction accuracy can be reached with medium-density SNP and with large data, even when QTL are not identified. Additionally, they found a significant disparity in the ability to identify CVs between populations with small *N_e_* (e.g., 60) and those with large *N_e_* (e.g., 600). In fact, populations with smaller *N_e_* required three times the number of genotyped animals with phenotypic data compared to larger *N_e_* populations. Despite these efforts, the study found that not all simulated QTNs were identifiable across varying *N_e_* or data intensities. The challenges in small populations primarily stem from longer chromosome segments, which complicate pinpointing the exact QTN location and increase uncertainty. Moreover, higher levels of noise can mask the true signal, making association detection difficult. The introduction of sequence data exacerbates these challenges, as achieving high-resolution GWAS becomes notably difficult under such conditions. These findings highlight critical considerations for genetic studies, particularly in the context of population size and data resolution. Certainly, the use of more genotyped animals and SNPs brings additional computational burden. However, a larger number of genotyped animals offers substantial benefits to GWAS: more detected QTLs (their number increases with approximately sqrt(*n*)) and more precise mapping of detected QTLs. Therefore, increasing the GWAS sample size clearly appears worth the additional computation cost.

## SNP Pruning of Sequence Data and Their Use in Genomic Prediction

The genome sizes of *Bos taurus* (domestic cattle)^1^ and *Ovis aries* (domestic sheep)^2^ are 2.8 Gb and 2.7 Gb, respectively. Some studies have shown that these nucleotides contain 36 million variants in cattle (Veerkamp et al., 2016). However, the use of such a large number of markers in genomic prediction with the current methodology, GBLUP, becomes infeasible, firstly, because building a **G** matrix with so many markers and several hundred thousand individuals routinely is very time-consuming, and secondly, because the **G** values show only a negligible change after the number of markers exceeds around 100K. Several strategies have been proposed to address these issues. One suggestion has been to select markers close to CVs or QTLs. This becomes more important in multibreed genomic prediction, where the LD and phase of markers and QTLs vary. The reliability of genomic prediction decreases as the distance between QTL and markers increases, especially in across-breed prediction (van den Berg et al., 2016). It has been suggested that eight requirements need to be met if a variant is to be considered as a CV. These include (1) using extensive and varied animal populations, (2) obtaining accurate genetic information, (3) measuring multiple observable traits, (4) providing detailed annotations of genomic regions, (5) conducting comparative analyses between species, (6) performing comparisons across entire genomes, (7) understanding the biological functions of potential genes, and (8) experimentally altering specific genomic sites (Meuwissen et al., 2022). CV selection can be based on the *P*-values of GWAS. However, this selection can be biased, as some markers in high LD with each other in some regions are likely to be selected (Veerkamp et al., 2016). This bias can be addressed by using the conditional and joint association analysis (COJO) method, which is designed to identify independent genetic variants associated with complex traits by accounting for LD between SNPs (Yang et al., 2012). However, the COJO method must be used cautiously, as it may cause over-fitting due to the large family structures in the data and collinearity between markers. The solution to this problem is explained in Veerkamp et al. (2016).

Meuwissen et al. (2024) showed that weighting SNPs in the **G** matrix process can increase genomic prediction reliability by 10% and 13% for single- and multi-trait analyses, respectively. This approach can improve the accuracy of genomic prediction after SNP pruning and weighting of CVs based on the GWAS results. In addition, the SNPs can be weighted based on the variance calculated from the Bayesian whole- genome regression model (Liu et al., 2020; Jang et al., 2023b).

Another approach is to add the CVs to the current chip, which has been shown to increase the reliability of prediction in dairy cattle (VanRaden et al., 2017), in sheep (Moghaddar et al., 2019), and in pigs (Jang et al., 2023b). Despite a possible increase in reliability (Liu et al., 2020), the rate of the increase was found to depend on the trait and the original chip. Building two **G** from the original chip and CVs is another approach to use CV to increase the reliability of genomic prediction accuracy (Al Kalaldeh et al., 2019; Moghaddar et al., 2019).

## Storing and Analyzing Large Datasets

Because solving large MME is computationally unfeasible, animal breeding scientists have designed algorithms capable of dealing with such MME for calculating BLUP for national genetic evaluations. These methods are based on iteration on data or out-of-core methods, which avoid the need to store MME coefficient matrices in the computer memory. The first iteration methods were developed already in the early 1980s (Schaeffer and Kennedy, 1986; Misztal and Gianola, 1988). Strandén and Lidauer (1999) presented a method known as the preconditioned conjugate gradient (PCG) for solving MME. They demonstrated its superiority over previous methods (e.g., Gauss-Seidel and Jacobi algorithms) in terms of its convergence properties and computational efficiency. The authors argued that PCG takes less time per iteration and requires fewer iterations to reach convergence compared to the old methods. PCG makes it possible to devise efficient general-purpose breeding value estimation software where the model can be changed by the user of the data. Since then, PCG has emerged as the method of choice for large-scale national genetic evaluations (Tsuruta et al., 2001).

Single-step GBLUP is theoretically the best model for the practical genetic evaluation of data on phenotyped individuals with or without genomic information. In general, the four leading computational approaches are the standard ssGBLUP (Aguilar et al., 2010; Christensen and Lund, 2010), ssGBLUP with the algorithm for proven and young (Misztal et al., 2014), Woodbury formula-based ssGTBLUP (Mäntysaari et al., 2017), and explicit marker equation formulations or ssSNP-BLUP (Fernando et al., 2014; Liu et al., 2014). They all have their strong and weak points, which means that their use depends on the amount of genomic data available and the population in question (Mäntysaari et al., 2020; Misztal et al., 2020).

In the following, we provide a concise review of different ssGBLUP approaches in terms of computing efficiency and predictability with a focus on the advantages and drawbacks of each method.

## Standard ssGBLUP

Despite the dramatic decrease in genotyping costs, it continues to be too expensive to produce genotypes for all animals, and, thus, a large proportion of animals in a population are not genotyped. The ssGBLUP approach is an elegant genomic prediction method, which enables integration of pedigree (genotyped and non-genotyped animals), genomic, and phenotypic information using Henderson’s MME. In ssGBLUP, the genome-based **G** can be combined with the pedigree-based additive relationship matrix **A** into a unified relationship matrix **H** (Aguilar et al., 2010; Christensen and Lund, 2010; Legarra et al., 2009).

Assume that animals are divided into genotyped (1) and non-genotyped (2) animals, the **A** matrix can be written as:


$$Α\text{=}\left[\begin{array}{l} A_{\text{11}}A_{\text{12}}\\ A_{\text{21}}A_{\text{22}} \end{array}\right],$$

The matrix **H** can be regarded as a modified matrix of **A** to accommodate **G,** and can be written as:


$$H=\left[\begin{array}{l} A_{11}-A_{12}A_{22}^{-1}A_{21}+A_{12}A_{22}^{-1}GA_{22}^{-1}A_{21}A_{12}A_{22}^{-1}G\\ GA_{22}^{-1}A_{21}\begin{array}{ccccccccccc} & & & & & & & & & & G \end{array} \end{array}\right],$$

and its inverse as:


$$H^{-1}=A^{-1}+\left[\begin{array}{l} 00\\ 0G^{-1}-A_{22}^{-1} \end{array}\right]$$

The **H** matrix is dense, whereas its inverse **H**^–1^ has a simple form. Solving the MME for ssGBLUP as presented in Aguilar et al. (2010) requires prior computation of the inverses of **G** and **A**_22_, which is not feasible in the case of a large number of genotyped animals. Moreover, the product ${\text{G = Z}}_{\text{c}}{\text{Z}}_{\text{c}}{}^{\prime }$ is not of full rank when the number of genotyped animals exceeds the number of SNPs, which has become a reality in many dairy or beef cattle populations. Thus, in order to achieve full rank, some ad hoc adjustments (as termed by Rohan Fernando) are required, such as adding small values to the diagonal elements of **G**, which is often singular, or combining **G** with the **A**_22_ matrix, that is, **G***_w_* = (1 – *w*)**G** + w**A**_22_ (VanRaden, 2008). It is worth noting that the addition of *w*, commonly known as the RPG weight, is justified because SNP markers cannot explain 100% of the total variance due to incomplete LD between SNPs and QTLs. The blending of **G** and **A**_22_ may thus improve the predictive ability of ssGBLUP (Fernando et al., 2014; Mäntysaari et al., 2020).

Inverting **G***_w_* for 222,619 genotyped animals using 10 CPU cores can take as much as 18.6*h* (Ben Zaabza et al., 2020). Given that the inversion of **G**_w_ increases cubically O(*n*^3^) with the number of genotyped animals, extrapolating to 1,125,000 genotyped animals would increase the computing time for the **G_w_** matrix inversion to almost 100*d* and require almost 10,125 GB of RAM. Although computing time for such a large number of genotyped animals can be reduced somewhat by using more powerful computers and more CPU cores, it is evident that when the number of genotyped animals exceeds 1 million, computing and inverting **G** becomes impossible or prohibitively expensive. This has prompted animal breeding scientists to develop other computational solutions and alternative approximation methods.

## The Algorithm for Proven and Young

Misztal et al. (2014) derived the algorithm for proven (i.e., with phenotypes and progeny) and young animals (APY) which uses recursions on a fraction of the genotyped animals to obtain a sparse representation of the inverse of **G**, herein-after called ${\text{G}}_{\text{APY}}^{–1}$, assuming that they represent most of the independent chromosome segments in the genome (Tsuruta et al., 2021). Because the original terms “proven” and “young” have become misleading, most publications now refer to them as “core” and “noncore” animals, respectively.

The proposed ${\text{G}}_{\text{APY}}^{–1}$ can be written as:


$$G_{\text{APY}}^{-1}=\left[\begin{array}{l} I-P_{\text{cn}}\\ 0\text{I} \end{array}\right]\left[\begin{array}{ll} G_{\text{cc}}^{-1} & 0\\ 0 & M_{\text{nn}}^{-1} \end{array}\right]\left[\begin{array}{ll} I & 0\\ -P_{\text{nc}} & \text{I} \end{array}\right]=\left[\begin{array}{ll} G^{\text{cc}} & G^{\text{cn}}\\ G^{\text{nc}} & M_{\text{nn}}^{-1} \end{array}\right],$$

where the subscript *c* stands for core animals and n for noncore animals; ${\text{P}}_{\text{nc}}={\text{G}}_{\text{nc}}{\text{G}}_{\text{cc}}^{–1}$ and ${\text{M}}_{\text{nn}}={\text{diagonal(G}}_{\text{nn}}–{\text{G}}_{\text{nc}}{\text{G}}_{\text{cc}}^{–1}{\text{G}}_{\text{cn}})$.

The computing cost of ${\text{G}}_{\text{APY}}^{–1}$ is cubic for core genotyped animals and linear for noncore genotyped animals, whereas the corresponding memory requirement is quadratic for core genotyped animals and linear for noncore animals. Thus, an increase in the number of genotyped animals increases linearly the number of computations in ${\text{G}}_{\text{APY}}^{–1}$ (Fragomeni et al., 2015).

The study by Misztal et al. (2014) was an overture to a series of papers, such as Lourenco et al. (2015), Pocrnic et al. (2016b), and Misztal (2016), which aimed at finding optimal parameters for APY, such as the number of core animals, choice of core animals, and the dependency of the number of core animals on a given trait. Misztal (2016) explained that the APY method is scalable even to very large data, especially for commercial livestock populations, which typically have a small *N_e_*, because a small core set will cover almost all genetic variation. The author demonstrated that when the number of core animals is as large as the number of independent chromosome segments, that is, from 10K to 15K in cattle populations, **G**^−1^ can be easily computed, and the computing time is substantially reduced. It is worth noting that he computation of ${\text{G}}_{\text{APY}}^{–1}\text{d}$ involves almost 2*cn_c_* + *c*^2^ + *n_c_* flops, where *c* is the number of core animals, and *n* is the number of noncore animals. Clearly, the cost of the ${\text{G}}^{\underline{c}}\begin{array}{c} 1\\ \text{APY} \end{array}\text{d}$ product is lower than that of **G**^−1^**d**, which requires *n*^2^ flops, where *n* = *n_c_* + *c* is the number of genotyped animals.

## ssGTBLUP Approach

Using the Woodbury formula, Mäntysaari et al. (2020) demonstrated that ${\text{G}}_{\text{w}}=(1–\text{w})\;Z_{\text{c}}Z_{\text{c}}{}^{\prime }+\text{w}A_{22}$ can be written as:


$$G_{\text{w}}^{-1}=\frac{1}{\text{w}}A_{22}^{-1}-\frac{1}{{\text{w}}^{2}}A_{22}^{-1}Z_{\text{c}}{\left(\frac{1}{\text{w}}Z_{\text{c}}{}^{\prime }A_{22}^{-1}Z_{\text{c}}+\frac{1}{1-\text{w}}I\right)}^{-1}Z_{\text{c}}{}^{\prime }A_{22}^{-1},$$

which can be written as Gw–1=1wA22–1–Tw′ Tw,

where $T_{\text{w}}=\frac{1}{\text{w}}L\begin{array}{c} –1\\ \text{w} \end{array}Z_{\text{c}}{}^{\prime }A_{22}^{–1}$, and the lower triangular matrix **L**_w_ is the Cholesky decomposition of $\frac{1}{\text{w}}Z_{\text{c}}{}^{\prime }A_{22}^{–1}Z_{\text{c}}+\frac{1}{1–\text{w}}I$ The method was named ssGTBLUP and has proved to be less computationally demanding than the regular ssGBLUP. The authors showed that their method is particularly useful when the number of genotyped animals is more than twice that of SNP markers.

Solving the MME for standard ssGBLUP with a PCG requires computing the product of **G**^−1^**d**, which requires *n*^2^ flops. In contrast, $G_{\text{w}}^{–1}\text{d = }$implies the computation of 2 terms: **T**_w_  **T**_w_**d** and the $\frac{1}{\text{w}}A_{22}^{–1}\text{d}$. The **T**_w_  **T**_w_**d** term requires 2*nm* flops, whereas the $\frac{1}{\text{w}}A_{22}^{–1}\text{d}$ can be rapidly computed using the method of Strandén et al. (2017), who showed that $A_{22}^{–1}\text{d}$ can be computed as **[A**^22^  **− A**^21^**(A**^11^) ^−1^**A**^12^**]d,** which only involves four sparse submatrices—**A**^22^, **A**^21^, and **A**^12^, and the inverse of A11. The authors presented 3 methods for avoiding the inversion of **A**^11^. For more details, see Strandén et al. (2017) and Mäntysaari et al. (2017). Thus, given the cost of computing $\left(T_{\text{w}}{}^{\prime }\;{\text{T}}_{\text{w}}\text{d}\right)\sim 2nm$, the ssGTBLUP model is particularly useful when *n* >> *m*, because the cost of computing $T_{\text{w}}{}^{\prime }\;{\text{T}}_{\text{w}}\text{d}$ is linear, while the cost of (**G**^−1^**d)** in regular ssGBLUP is quadratic in terms of *n*.

The original ssGTBLUP approach has an important drawback. The **T**_w_ matrix has the size of *m* by *n*, increasing the memory requirements as the number of genotyped *n* increases, and having as many rows as the number of SNP markers *m*. To address this challenge, a component-wise ssGTBLUP approach was developed based on the formula (Mäntysaari et al., 2020; Vandenplas et al., 2023):


$$G_{\text{w}}^{-1}=\frac{1}{\text{w}}A_{22}^{-1}-\frac{1}{{\text{w}}^{2}}A_{22}^{-1}Z_{\text{c}}KZ_{\text{c}}{}^{\prime }A_{22}^{-1}$$

where $K={\left(\frac{1}{\text{w}}Z_{\text{c}}{}^{\prime }A_{22}^{–1}Z_{\text{c}}+\frac{1}{1–\text{w}}I\right)}^{–1}$. In the component-wise approach, only the K matrix needs to be precomputed. $G_{\text{w}}^{–1}\text{d}$ needed in PCG is done in steps from the innermost parenthesis outward in ${\text{G}}_{\text{w}}^{–1}\text{d=}\frac{1}{\text{w}}A_{22}^{–1}\text{d-}\frac{1}{{\text{w}}^{2}}A_{22}^{–1}\left(Z_{\text{c}}\left(\text{K}\left(Z_{\text{c}}{}^{\prime }\left(A_{22}^{–1}d\right)\right)\right)\right)$. The original marker matrix Z is stored efficiency by packing five genotypes to a byte, and the centering for the Zc matrix is done in-the-fly. The Z_c_ and $A_{22}^{–1}$ computations can be made efficiently using parallel computing. The component-wise ssGTBLUP allows reducing the amount of memory from the original 8(*nm + m*2) bytes to 8*m2 + nm*/5 bytes when double precision is used to store the K matrix. For example, when n is 1 million and *m* is 50,000, the amount of memory required is reduced from about 420 GB to 30 GB. Using less memory results in more efficient use of cache memory and more efficient computations. For example, in Vandenplas et al. (2023), the number of genotyped was 2.61 million and the number of SNP markers was 47,006, resulting in 172.4 s and 64.9 s per iteration for the original and the component-wise ssGTABLUP approach, respectively.

## Single-Step Marker Models

When the number of genotyped animals grows extremely large, ssGBLUP models appear to reach their limits, and thus, using single-step marker models, which are limited by the number of markers, can provide computationally efficient solutions, such as those proposed by Fernando et al. (2014). The authors presented a strategy for Bayesian regression models (SSBR). Their strategy combines all available data from genotyped and non-genotyped animals, as in ssGBLUP, but is suitable for a variety of models. Marker covariates are imputed for non-genotyped animals, and a residual genetic effect is added to account for deviations between true and imputed genotypes.

The MME for the SSBR is


$$\left[\begin{array}{l} X^{\prime }R^{-1}X\begin{array}{cc} & \begin{array}{ccc} & & X_{1}{}^{\prime }R_{1}^{-1}Z_{1} \end{array} \end{array}\\ M^{\prime }Z^{\prime }R^{-1}XM^{\prime }Z^{\prime }R^{-1}ZM+I\frac{1}{\sigma_{\text{g}}^{2}}M_{1}{}^{\prime }Z_{1}{}^{\prime }R_{1}^{-1}Z_{1}\\ Z_{1}{}^{\prime }R_{1}^{-1}X_{1}Z_{1}{}^{\prime }R_{1}^{-1}Z_{1}M_{1}\begin{array}{cc} & \begin{array}{cc} & Z_{1}{}^{\prime }R_{1}^{-1}Z_{1}+A^{11}\frac{1}{\sigma_{\text{a}}^{2}} \end{array} \end{array} \end{array}\right]\left[\begin{array}{c} \hat{\text{b}}\\ \hat{\text{g}}\\ \hat{\text{ε}} \end{array}\right]\text{=}\left[\begin{array}{l} X^{\prime }R^{-1}\text{y}\\ M^{\prime }Z^{\prime }R^{-1}\text{y}\\ Z_{1}{}^{\prime }R_{1}^{-1}{\text{y}}_{1} \end{array}\right],$$

where **M** is the matrix of imputed and observed genotypes of all animals in the pedigree, **M**_1_ is a submatrix of imputed genotypes of non-genotyped animals with phenotype, and b̂, ĝ, and *ε̂* are solutions for fixed effects, marker effects, and imputation residual effects, respectively. These authors continued their work to derive the hybrid model (Fernando et al., 2016). The MME of the standard ssGBLUP is quite different from these models, and, as a result, the SSBR and the hybrid models are rarely available in genetic prediction software. A more widely used single-step marker model is that of Liu et al. (2014), often referred to as ssSNPBLUP. The MME of ssSNPBLUP is similar to an animal model augmented with marker information, and there is no need to build a genomic relationship matrix, making the preprocessing step very fast. When the marker effects in the MME of the ssSNPBLUP model are absorbed into the other effects, the resulting MME is the same as ssGTABLUP. Despite the similarity, ssSNPBLUP has lower preprocessing costs but poorer convergence properties than ssGTABLUP (Vandenplas et al., 2023).

A general pattern that emerges from comparing the available approaches is that small datasets can be handled efficiently enough with the standard ssGBLUP, and that ssGTABLUP is fast for medium-size data, but ssSNPBLUP and APY ssGBLUP for large data. A problem with ssSNPBLUP is that, unlike APY, it may become computationally more challenging as the number of markers increases. The iterative solving convergence is poorer in SNP-BLUP than GBLUP-type models and deteriorates with the increase in the number of markers. Therefore, using sequence data may be prohibitive because the efficiency of SNP-BLUP-based methods is bounded by the number of SNPs. However, there are many marker-based models, and they differ in efficiency and iterative solving convergence characteristics (Taskinen et al., 2017), and some even allow APY-like data reductions on chromosome level (Ødegård et al., 2018), making a general conclusion impossible.

## Genomic Breeding Value Reliability Computations

The increase in genomic information has exacerbated the challenges involved in calculating the reliability of EBV (Ben Zaabza et al., 2023). Individual EBV reliability depends on the amount of information that contributed to that prediction. Calculating the exact reliability of EBV requires determining the prediction error variance (PEV), which is derived from the elements of the inverse of the MME coefficient matrix. The computation of the inverse becomes infeasible in the case of a large MME. In the animal model, the size of the MME is equal to the number of levels of fixed and random effects in the model. For large pedigree-based animal models, computing reliabilities using an exact approach is practically impossible. The need to find alternative computational solutions has led to the development of several animal model approximation methods to PEV that avoid the MME coefficient matrix inversion (Misztal and Wiggans, 1988; Liu et al., 2004, 2010; Tier and Meyer, 2004; Ducrocq and Schneider, 2007).

Genomic selection was adopted very quickly after its first implementation in 2009 (VanRaden, 2020). The entire genomics field has benefited from the increased availability of genomic data. The two equivalent genomic models called SNP-BLUP and GBLUP are widely used in genomic selection. It is worth noting that equivalence here means that two models have equal phenotypic variance and their fixed effect predictions are also equal. Thus, they yield equal EBV and PEV at the animal level.

In SNP-BLUP, the size of the MME increases with the number of SNP markers m, whereas in the GBLUP model, the size of the MME increases with the number of genotyped animals *n*. Moreover, the individual EBV reliability of GBLUP model requires setting up and inverting the genomic relationship matrix **G** before inverting the MME coefficient matrix. This can represent a heavy computational burden, especially when dealing with databases for millions of genotyped animals.

It has been shown that tens of thousands of SNPs can be sufficient to perform genomic predictions with high prediction accuracy. Commonly used SNP arrays vary in their content, but usually contain around 50,000 SNPs. With the availability of inexpensive SNP arrays, the number of genotyped animals has increased dramatically. For example, in the United States, *n* has increased from thousands in 2009 (first implementation of genomic selection) to several million in 2024. Overall, the number of SNP markers is more stable than the number of genotyped animals. This indicates that SNP-BLUP models are computationally scalable when the number of genotyped animals increases. For instance, in the case of 500,000 animals genotyped using 50,000 SNPs, the computing time required to invert the MME is approximately proportional to (500,000/50,000)^3^ = 1,000 for GBLUP over SNP-BLUP. LD between SNPs and QTL is known to be incomplete, and a portion of the total variation remains unexplained. Therefore, the inclusion of RPGs in the SNP-BLUP model can improve its predictive ability by capturing the proportion of variance not captured by SNPs (Liu et al., 2016; Ben Zaabza et al., 2020). However, calculating the individual EBV reliabilities in an SNP-BLUP model when RPGs are included would increase the size of the MME by the number of genotyped animals, and the MME inversion would thus increase cubically with O((*m* + *n*)^3^) instead of O(*m*^3^). This is computationally more expensive than in GBLUP models. Ben Zaabza et al. (2020) proposed a Monte Carlo–based approach, termed MC-SNPBLUP, to approximate the EBV reliabilities in a SNP-BLUP model when RPG is included. The method allows reducing the number of RPG from n to the number of MC samples *n*_mc_, which is always less than *n*. More specifically, MC samples are used instead of the Cholesky decomposition of the pedigree-based relationship matrix of genotyped animals **A**_22_; that is, **A**_22_ = **LL′**. Later, Ben Zaabza et al. (2021) extended their method to the use of MC samples for both RPG and SNP marker effects in a method called Full-MC-SNP-BLUP, where the size of the MME becomes *n*_mc_ instead of (*n*_mc_ + *m*) and, for example, the computational cost of MME inversion increases cubically with the number of samples ~ O[(*n*_mc_)^3^)]. This method has been shown to be computationally efficient. However, it suffers from a lack of precision (reliability inflation) for animals with low reliability, especially when using high RPG effect weights. Conceptually, Full-MC-SNP-BLUP could be applied to sequence data. However, this would most likely require a large number of MC samples, which may limit its efficiency. Gao et al. (2023) proposed an index-based approximation that combines EBV reliabilities from a SNP-BLUP model without RPG with reliabilities from a pedigree-based animal model. This approach limits the computational cost of MME inversion in the SNP-BLUP model to *m*. When sequence data is analyzed, the EBV reliabilities of the SNP-BLUP model without RPG may need to use the Monte Carlo approach for the marker data, as in Ben Zaabza et al. (2021), because the MME of the SNP-BLUP model becomes too large.

## EBV Reliabilities for Single-Step Genomic Models

The reliability of predictions from an ssGBLUP model inherits the challenges faced in calculating reliabilities for pedigree-based animal and multistep genomic models (Ben Zaabza et al., 2023). The calculation of exact ssGBLUP model reliabilities requires the construction and subsequent inversion of the MME coefficient matrix. The size of the MME increases with the number of levels of fixed effects and the number of animals in ssGBLUP, which becomes computationally demanding as the number of pedigree animals increases. Moreover, the need to make and invert the **A**_22_ and **G** matrices in advance further increases this challenge. Despite the availability of powerful computers, it is evident that calculating exact ssGBLUP reliabilities for SNP data amounts, pedigree, and phenotype databases for millions of animals is impossible. Several approximation methods have been proposed to overcome this problem. Misztal et al. (2013) presented two approximation methods based on the decomposition of PEV into contributions from pedigree, phenotype, and genomic information, in terms of effective daughters or observations. For more details, see Misztal et al. (2013). An advantage of the first method is its high precision, that is, the high correlation between exact ssGBLUP reliabilities and approximation method (*r* = 0.98). A drawback of this method is the need to explicitly invert the **A**_22_ and **G** matrices, which poses a computational burden in the case of a large number of genotyped animals. The second method, on the other hand, is less computationally demanding as it ignores the off- diagonals of the inverses, but this comes at the expense of precision (*r* = 0.72). The authors showed that both approximation reliability methods are efficient and accurate, as long as the number of genotyped animals remains under 100,000.

Later, multistep methods for estimating the EBV reliabilities in ssGBLUP models without inverting the ssGBLUP MME have been proposed, for example, by Liu et al. (2017), Edel et al. (2019), Ben Zaabza et al. (2022), and Gao et al. (2023). These methods are quite similar but differ in details. The methods are based on estimating the amount of non-genomic information for genotyped animals in the pedigree-based animal model, and using this non-genomic information to obtain the total information in a genomic model and the ssGBLUP reliabilities for the genotyped animals. Finally, the increased information due to the genomic data for the genotyped animals is used as weights in the pedigree-based animal model to approximate the reliabilities for the non-genotyped animals in ssGBLUP. These methods have proven to be efficient even when dealing with large datasets. However, when using sequence data implying millions of SNPs, the methods will have the same limitations as already described for SNP-BLUP. For example, in Liu et al. (2017), the EBV reliabilities for genotyped animals are calculated by a pure SNP-BLUP model where the size of the MME is limited by the number of SNPs m. Similarly, in Ben Zaabza et al. (2022), the genomic EBV reliabilities for the genotyped animals were approximated by an MC-SNP-BLUP model. Moreover, the use of sequence data would most likely require many more MC samples, which could increase the computational burden. Similar to Liu et al. (2017), Berman et al. (2022) developed a multistep method to approximate the EBV reliabilities in ssGBLUP. The main difference is the use of sparsity of ${\text{G}}_{\text{APY}}^{–1}$ in the computation of PEV in GBLUP. The authors showed that PEV can be written as the diagonal of the inverse of $\left({\text{D+G}}_{\text{APY}}^{–1}\right)$, where the diagonal **D** matrix has weights. The APY-based algorithm for approximating PEV in GBLUP can be extended to single- and multiple- trait ssGBLUP model EBV reliabilities by using a technique based on effective record contributions (Berman et al., 2022). The choice of appropriate core animals can affect the success of the APY-based method. Several approximation methods have been proven successful in dealing with large datasets involving millions of genotyped animals and standard SNP chips, where *n* >> *m*. Some of these methods use SNP-BLUP as an intermediate step to calculate the genomic EBV reliabilities for genotyped animals. However, in the case of large sequence data, where both m and n can be in the millions, it is clear that exact SNP-BLUP model approaches cannot be recommended. Thus, the best strategy to handle any large data with any SNP densities would be to develop approximations, such as the use of only a subset of markers, and improve the existing algorithms developed by animal breeding scientists.

## Computer Resources Requirements for Dealing With Sequence Data

Analyzing sequence data requires substantial computing resources due to the vast amount of data involved and the complexity of the analyses. Programs need to be optimized and developed to utilize the available computing power efficiently. The slowest hardware component will often represent a bottleneck. Below we delve into the details of four key computer components: Central Processing Unit (CPU), GPU, Random Access Memory (RAM), and storage devices, and describe their role in sequence data analysis.

## Central Processing Unit

High clock speeds, multiple CPU cores, and vectorization are essential tools that underline the significance of the work of researchers and data scientists in processing large datasets efficiently. While the number of CPU cores and their speeds are critical factors for efficiency, utilizing vectorization capabilities within the CPUs can significantly improve the performance. For example, the Advanced Vector Extensions 512 (AVX-512) can process sixteen 32-bit integers simultaneously (Cebrian et al., 2020). The use of multiple CPU cores, vectorization, and other techniques, such as memory alignment, contributes to the high performance of libraries such as Intel’s Math Kernel Library and OpenBLAS (Zhang et al., 2011). The well-known, efficient program Plink (Chang et al., 2015) uses multiple cores to analyze genomic data. Additionally, multiple servers can be utilized, and some programs make it possible to distribute the process across multiple computers. Libraries such as the Message Passing Interface (MPI) and Hadoop can be used for this purpose (Clarke et al., 1994; O’Driscoll et al., 2013).

## Graphics Processing Unit

GPU programming is highly effective for analyzing sequence data due to its ability to perform massively parallel computations, which significantly speed up processing times compared to CPU-based approaches. CUDA (Luebke, 2008) is a parallel computing platform and programming model developed by NVIDIA. However, programming on this platform limits the analysis to NVIDIA GPUs. Standards like Open Computing Language (OpenCL) and interfaces like OpenMP (Ayguadé et al., 2009; Munshi, 2009) can be used not only to exploit the power of GPUs, but simultaneously that of CPUs and other GPUs from AMD and Intel. GPU-accelerated sequence analysis has been used, for example, for sequence alignment (Ahmed et al., 2019), epistasis detection (Wienbrandt et al., 2023), haplotype and genotype imputation (Chen et al., 2012), and a comprehensive suite for processes such as quality control, alignment, and variant calling in NVIDIA Parabricks (NVIDIA, 2019).

## Random Access Memory

RAM is a critical component in both personal computers and servers. However, the types of RAMs used in these systems can differ significantly depending on their specific requirements and workloads. The main difference is that the server RAM supports the Error-Correcting Code (ECC), which can detect and correct data corruption (Krishnan et al., 2009). ECC is essential for servers where data integrity and uptime are critical, although it may slightly decrease performance. ECC is a crucial bonus in the server RAMs, as sequence data analysis can take several weeks to complete.

When analyzing large sequence data, the amount of RAM is the most important factor. The main RAM memory is supported by a small high-speed cache memory, which acts like a buffer between the RAM and the CPU. When a lot of data needs to be extracted from RAM, accessing the RAM through cache can become a bottleneck and slow down the computations. Thus, RAM is often used most efficiently by minimizing the amount used. For example, the high speed of Plink comes partly from packing multiple markers into one byte, which allows more markers to be in the cache memory than without packing. Furthermore, unpacking of data in Plink is efficient, and packing allows large sequence data to be in RAM.

The amount of RAM needed to analyze genomic data can be estimated prior to analysis; this depends highly on the programs used; however, as some programs like FImpute (Sargolzaei et al. 2014) use virtual memory internally. Two other memory criteria are memory speed and Column Address Strobe (CAL) Latency (CL). The first denotes the memory speed, and the second indicates the delay time between the memory controller requesting data and the available data; therefore, the lower the CL, the faster the memory. These can affect the performance of the program in combination with the CPU clock speed.

## Storage Devices

Several storage devices are available for storing sequence data. The fastest storage device currently available is nonvolatile memory express (NVMe), known for its high-speed data processing capabilities. These storage devices, though expensive, are ideal for the operating system and temporary storage of sequence data that needs to be analyzed. Solid state drive (SSDs) and hard disk drive (HDDs), on the other hand, are suitable for long-term data storage. SSDs, which are nearly 4 to 5 times faster than HDDs, are almost four times more expensive per kilobyte of storage. Another storage option is a tape drive, which is slow and costly to set up, but offers a cost-effective solution for backing up sequence data. Tapes are less expensive than HDDs, making them a prudent choice for long-term data storage (Batley and Edwards, 2009).

## Final Considerations

The advent of genomic-scanning technologies and relatively inexpensive genotyping and sequencing has led to increased availability of genomic data, both in terms of the number of animals genotyped and variants detected/SNP marker density. This has created new computational challenges, especially related to the use of sequence data. Animal breeding scientists have succeeded in developing efficient algorithms and programs capable of handling ever-growing datasets, including genomic sequence data. However, there is still a need to revise and improve the computational methods and algorithms for higher efficiency. The analysis of sequence data requires substantial computing resources as the amount of genomic data increases. Programs need to be optimized and further developed to fully harness the available computing power. Initially, the introduction of sequence data raised high hopes for major gains in genomic prediction accuracy over standard SNP arrays. However, the use of sequence data has shown either no gain or only a limited gain in prediction accuracy, particularly in populations of small effective size such as cattle. It is noteworthy, however, that such results may vary depending on the model assumptions (i.e., QTL distributions), statistical methods used in the analysis (GBLUP vs. Bayesian approaches), data being analyzed (simulations vs. real data), and demographic history and population structures. Clearly, sequence data should not be used directly for genomic prediction, but instead, for the identification of putative CVs to improve the accuracy and stability of subsequent genomic predictions. The growing number of genotyped animals also presents a challenge, and it is therefore crucial to use a set of core animals for genomic evaluation rather than including all reference animals. An alternative method could be an SNPBLUP-based approach, which is efficient since it is bounded by the number of used SNP markers.

## Footnotes

1 Genome assembly ARS-UCD2.0, https://www.ncbi.nlm.nih.gov/datasets/genome/GCF_002263795.3/2ARS-UI_Ramb_v3.0, https://www.ncbi.nlm.nih.gov/datasets/genome/GCF_016772045.2/).

2 ARS-UI_Ramb_v3.0, https://www.ncbi.nlm.nih.gov/datasets/genome/GCF_016772045.2/).

## Abbreviations:

**A**: pedigree-based relationship matrix
**A_22_**: pedigree-based relationship matrix of genotyped animals
APY: algorithm for proven and young animals
BLUP: best linear unbiased prediction
CAL: column address strobe
CNV: copy number variation
COJO: conditional and joint association analysis
CPU: central processing unit
DGS: DiGeorge syndrome
ECC: Error-Correcting Code
GATK: Genome Analysis Toolkit
GBLUP: genomic BLUP
GEBV: genomic EBV
GPU: Graphics Processing Unit
GWAS: genome-wide association studies
*h* ^2^: heritability of the trait
HDD: hard disk drive
INDELs: insertions and deletions
LD: linkage disequilibrium
LPS: low-pass sequencing
MAF: minor allele frequency
M_e_: number of independent chromosomes segments
*N*_e_: effective population sizes
NVMe: nonvolatile memory express
ONT: Oxford Nanopore Technologies
OpenCL: open computing language
PacBio HiFi: Pacific Biosciences High Fidelity
PCG: preconditioned conjugate gradient
PCs: personal computers
PEV: prediction error variance
PLD: pairwise linkage disequilibrium
PRS: polygenic risk scores
QTL: quantitative trait loci
RAM: random access memory
RPG: residual polygenic effects
SNP: single-nucleotide polymorphisms
ssSNPBLUP: single-step SNP-BLUP
SNVs: single-nucleotide variants
SSBR: single-step Bayesian regression
SSD: solid state drive
ssGBLUP: single-step GBLUP
ssGTBLUP: single-step GBLUP by the Woodbury formula
SVs: structural variations
WGS: whole-genome sequence
